# Effect of hydrogen gas inhalation on patient QOL after hepatectomy: protocol for a randomized controlled trial

**DOI:** 10.1186/s13063-021-05697-5

**Published:** 2021-10-21

**Authors:** Masaki Kaibori, Hisashi Kosaka

**Affiliations:** Department of Surgery, Kansai Medical University, Hirakata, Japan

**Keywords:** Hydrogen inhalation, Hepatocellular carcinoma, QOL, Hepatectomy

## Abstract

**Introduction:**

Molecular hydrogen had been considered inactive in vivo but is an antioxidant that selectively reduces highly toxic reactive oxygen species (ROS). Animal studies have reported that hydrogen gas inhalation helped alleviate cerebral and cardiac ischemia-reperfusion injuries. In humans, hydrogen inhalation therapy is presently approved as a treatment under Advanced Medical Care B in Japan (jRCTs031180352: limited to adult patients who suffered out-of-hospital cardiac arrest and are in a continuous coma) and its effectiveness is being examined in a clinical trial. The Japanese government has introduced the “Advanced Medical Care System” to promote the development of drugs and devices under governmental regulations. Advanced Medical Care B is a system designed for unapproved or off-label drugs or medical technologies used in a clinical trial setting. Hepatectomy is generally performed with repeated hepatic blood-flow occlusion and then reperfusion (ischemia and reperfusion). No report, however, has been made on ROS inhibition by hydrogen inhalation therapy or its effectiveness in post-hepatectomy patients. Hydrogen gas inhalation in the early stages after hepatectomy is anticipated to inhibit liver dysfunction by inhibiting ROS.

**Methods and analysis:**

This study is a randomized, controlled, double-blind superiority trial, which will be conducted as a “specified clinical trial” in accordance with the Clinical Trials Act in Japan. Trial registration was prospectively completed before the first participant was enrolled.

The subjects will be patients who will undergo hepatectomy and will be allocated randomly into group A with hydrogen gas inhalation or group B with air inhalation after hepatectomy. The study will examine if hydrogen gas inhalation improves QOL of post-hepatectomy patients. The primary endpoint is patient QOL (score of a 40-item quality of recovery questionnaire, QoR40) on postoperative day 3 and the secondary endpoints are QoR40s besides that on postoperative day 3, grade of postoperative complications (Clavien-Dindo score), level of pain (Numerical Rating Scale (NRS)), amount of dietary intake, liver function, inflammation level, 8-hydroxydeoxyguanosine (urinary 8-OHdG) level, and number of pedometer-assessed steps.

**Ethics and dissemination:**

The study protocol has been approved by the Niigata University Central Review Board of Clinical Research. The findings of this study will be widely disseminated through peer-reviewed publications and conference presentations.

**Trial registration:**

jRCTs 03220332. Registered on 21 January 2021

## Strengths and limitations of this study

・The present double-blind RCT is expected to show that early clinical implementation of hydrogen gas inhalation after hepatectomy will inhibit ROS, thereby inhibiting liver dysfunction.

・Hydrogen gas inhalation could also be shown to contribute to early recovery of post-hepatectomy patients.

## Introduction

Molecular hydrogen had been considered inactive in vivo but is an antioxidant that selectively reduces highly toxic reactive oxygen species (ROS). In 2007, a rat study reported that hydrogen gas inhalation alleviated cerebral ischemia-reperfusion injuries [1]. Focal ischemia was produced by mild occlusion of the middle cerebral artery for 90 min, followed by reperfusion for 30 min. The rats inhaled 1–4% hydrogen gas mixed with inhalation anesthetics during the entire 120-min process. After 1 day, the infarct volume markedly decreased due to hydrogen gas inhalation. After 1 week, neurological symptoms improved, as indicated by improved motor function, and oxidative stress was reduced, as indicated by reduced 8-OHdG level near the infarct site.

There have been many reports on the inhibitory effect of hydrogen gas inhalation on ischemia-reperfusion injury in organs other than the brain [2, 3]. In rats, hydrogen gas inhalation reduced the cardiac infarct volume after coronary-artery ischemia and reperfusion, and cardiac function was protected [4]. Similar effects have also been reported in pigs [5]. Presently, hydrogen gas inhalation is approved as a treatment in Japan under Advanced Medical Care B (jRCTs031180352: limited to adult patients who suffered out-of-hospital cardiac arrest and are in a continuous coma), and its effectiveness is being examined in a clinical trial.

Hepatectomy is generally performed with repeated hepatic blood-flow occlusion and then reperfusion (ischemia and reperfusion). No report, however, has been made on ROS inhibition by hydrogen inhalation therapy or its clinical effectiveness in post-hepatectomy patients. Hydrogen gas inhalation in the early stages after hepatectomy is anticipated to inhibit liver dysfunction by inhibiting ROS. The present double-blind RCT is expected to show that early (i.e., after liver ischemia-reperfusion) clinical implementation of hydrogen gas inhalation after hepatectomy will inhibit ROS, thereby inhibiting liver dysfunction. Furthermore, hydrogen gas inhalation may be shown to contribute to early recovery of post-hepatectomy patients.

## Methods/design

This study is a prospective randomized, controlled, double-blind superiority trial which will be conducted as a “specified clinical trial” in accordance with the Clinical Trials Act in Japan. The study will involve 68 patients with hepatobiliary disease and who will undergo hepatectomy at Kansai Medical University Hospital in Japan. The subjects will be allocated randomly into group A (hydrogen gas inhalation group: 34 subjects) and group B (air inhalation group: 34 subjects). Groups A and B will receive 1200 ml/m hydrogen gas and 1200 ml/m air, respectively, via nasal cannula. The respective gas will be administered at least 1 h per time and three times per day, totaling 8 h or less. Air will be used as the placebo comparator to detect the effectiveness of hydrogen inhalation therapy. The air will have no odor, color, or additional effect on postoperative recovery. Moreover, post-hepatectomy patients are typically on room air. Face-to-face adherence reminder sessions will take place at the initial inhalation and at each study visit thereafter. This session includes notification of the total time count of the inhalation period and reinforcement that the study gas may be hydrogen gas or air. No concomitant treatments are prohibited. If a subject will require oxygen, it will be administered via an oxygen mask placed over the nasal cannula. The trial participation period starts after consent attainment and ends at postoperative hospital discharge. The period is expected to be approximately 14–21 days based on our previous experience. The 40-item quality of recovery questionnaire (QoR40) will be used to assess postoperative QOL [6]. The questionnaire will be distributed preoperatively to patients who will independently fill it out themselves. The results will be totaled (perfect score: 200 points) to evaluate postoperative patient satisfaction and recovery level (reference material in the appendix at the end of this paper). The questionnaire survey will be conducted at 10 AM on postoperative day 3.

The results of previous reports infer that hydrogen gas inhalation has wide effects on many aspects, such as on inflammation and liver function, by inhibiting ROS [1–5, 7–10]. The primary endpoint, therefore, is established as the QoR40 on postoperative day 3.

### Sample size estimation

In the field of this study, the QoR40 is constructed from 40 items related to “physical comfort,” “emotional state,” “physical independence,” “psychological support,” and “pain.” The maximum score per item is 5 points and the perfect total score, therefore, is 200 points [6]. In hemodialysis patients, it has been reported that symptoms of fatigue were reduced by approximately 30% using a dialysate containing dissolved molecular hydrogen [11]. Molecular hydrogen might affect particularly “physical comfort” and “emotional state” among the 20 items of the QoR40. The results will be considered clinically meaningful if hydrogen gas inhalation is to improve the items of “physical comfort” and “emotion state” by approximately 20%. If such improvement in scores occurs, the hydrogen gas inhalation group will be estimated to score approximately 16 points more than the air inhalation group and to have an average of approximately 153 points. When factors are considered, the estimated average of the hydrogen inhalation group will be 151 points, that of the non-hydrogen inhalation group will be 137 points, and the variance of data 30 points (same in both groups). If α-error and power are set at 0.20 and 70%, respectively, for a two-sided test, then 31 subjects per group (total of 62 subjects) will need to be enrolled in this study. If it is estimated that approximately 10% of the subjects will drop out, the target number of subjects will be 68.

### Recruitment

The recruited subjects will be patients who provide a written consent of their own free will to participate after being fully informed of and fully understanding the study. At Kansai Medical University Hospital, between 10 and 15 patients undergo hepatectomy per month. Upon admission to Kansai Medical University Hospital, study participants will be recruited by surgeons participating in the study. The surgeon will obtain informed consent from the participant. Study subjects will receive no financial incentives for participation.

### Inclusion criteria

1. Male or female aged 20 or older at the time of consent
2. Patients with hepatobiliary disease and will be undergoing hepatectomy

- Laparotomic or laparoscopic hepatectomy
- Any extent of liver resection
- Any hepatobiliary disease (including hepatocellular carcinoma, intrahepatic cholangiocarcinoma, and metastatic liver cancer)

3. Patients who provide a written consent of their own free will to participate after being fully informed of and fully understanding the study

### Exclusion criteria

Patients who meet any of the following criteria will not be enrolled in this study:

1. Patients with severe liver dysfunction or severe kidney dysfunction (patients satisfying any of the following: total serum bilirubin > 3.6 mg/dL, AST > 175 U/L, ALT > 175 U/L, creatinine > 3 mg/dL, or eGFR < 30 mL/min/1.73m^2^)
2. Poorly controlled diabetes
3. Female patients who are or might become pregnant
4. Female patients who are lactating
5. Patients who received another test drug or investigational drug within 3 months of consent
6. Patients who might undergo resection of an organ besides the liver (including the stomach, colon, and lung but excluding the gall bladder) at the time of hepatectomy
7. Patients who will require biliary tract reconstruction at the time of hepatectomy
8. Patients who will undergo emergency surgery (including for ruptured liver tumor)
9. Patients whom the principal investigator determined to be unsuitable for the study

### Randomization and allocation concealment

After the research team obtains patient consent from the recruited participants, web-based registration and minimization method of random allocation will be performed at the data center of Medical Research Support, a company located in Japan that is independent of the research team. Medical Research Support will generate web-based randomization sequences. The research team cannot access the randomization process and will be informed of the results of the randomization via email from Medical Research Support. The minimization method of random allocation prevents the prediction of study allocation. Then, the registration numbers and the allocation results will be added to the eligibility criteria checklist. The information will be faxed to the data center, resulting in patient registration.

### Blinding

In this double-blind trial, the device that supplies air is manufactured so that it is indistinguishable by appearance from the hydrogen gas generator. The markers are pasted on the behind of generators to distinguish which generator is the hydrogen gas generator or the air generator. Only the principal investigator knows the meaning of marking, whereas the research stuff are not informed of the meaning of marking. The web-based patient allocation was known only to the person responsible for statistical analysis, the person in charge of support for the research and development program, and the principal investigator. However, research staff and patients cannot know the allocation. If unblinding is deemed to be necessary, such as for treatment of emergencies, the principal investigator should open the allocation. Unblinding is not necessarily a reason for discontinuation of study gas.

### Interventions and data collection

**Fig. 1:**
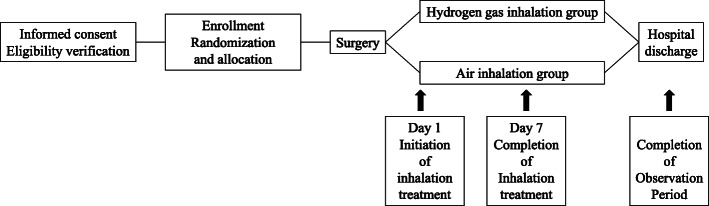
From postoperative day 1 to day 7, groups A and B (34 subjects each) will receive 5% hydrogen gas and air, respectively

From postoperative day 1 to day 7, groups A and B (34 subjects each) will receive 5% hydrogen gas and air, respectively, via nasal cannula for at least 1 h per time and three times per day, totaling 8 h or less (Fig. 1). The study schedule is summarized in Table 1. Upon patient enrollment, the study site staff will make every reasonable effort to follow the participant to ensure that a complete data set is obtained and to avoid associated complexities across the entire study period. All randomized participants who prematurely discontinue study gas inhalation will be considered to be out of the study and will follow the same schedule of events as those participants who continue study gas inhalation, except that the QoR40 and urinary 8-OHdG will not be assessed. All of these participants will be followed until discharge from the hospital as scheduled.

1. QoR40 scores: Subjects will fill out themselves the previously distributed questionnaire preoperatively, on postoperative days 3 and 7, and at discharge. The questionnaire will be collected by the attending nurse. The primary endpoint is the QoR40 on postoperative day 3.
2. Grade of postoperative complications (Clavien-Dindo score): The attending physician will evaluate on the day of discharge [12].
3. Level of pain (Numerical Rating Scale (NRS)): The attending nurse will record it in the medical record on postoperative days 1, 3, 5, and 7 and on the day of discharge.
4. Amount of dietary intake: The attending nurse will record it in the medical record preoperatively, on postoperative days 3, 5, and 7 and on the day of discharge.
5. Number of pedometer-assessed steps: The number of steps per day will be measured using a pedometer preoperatively and on postoperative days 3, 5, and 7. The attending nurse will record the numbers in the medical record.
6. Inflammatory response (white blood cell count, differential white blood count, and lymphocyte count): They will be measured preoperatively, on postoperative days 0, 1, 3, 5, and 7 and on the day of discharge. They will be automatically recorded in the medical record.
7. Liver function (T-BIL, AST, ALT, ALP, albumin, PT, and ICG): The ICG test will be performed only one time, namely preoperatively. Other measurements will be made preoperatively and on postoperative days 0, 1, 3, 5, and 7 and on the day of discharge. All measurements will be automatically recorded in the medical record.
8. Reactive oxygen level (urinary 8-OHdG level): A subinvestigator will measure preoperatively, on postoperative days 3 and 7 and on the day of discharge and then will record the measurements in the case report form.
9. Adverse events: Common Terminology Criteria for Adverse Events (CTCAE) version 5.0.
10. Duration of study gas inhalation (measured per 24 h and totaled by week)

**Table 1:**
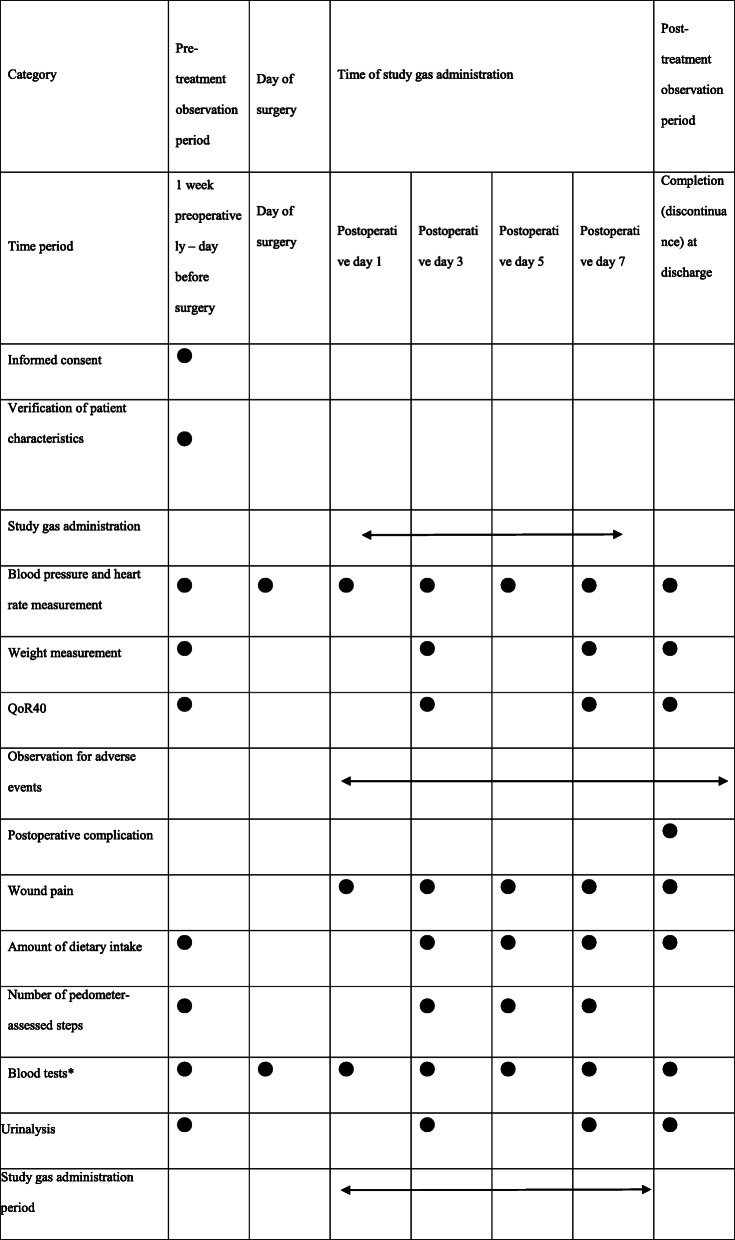
Study schedule

*QoR40* the 40-item quality of recovery questionnaire

Participant files are to be stored in numerical order in a secure and locked location. Participant files will be maintained in storage for 10 years after completion of the study.

#### Primary endpoint

The postoperative recovery level (QoR40) on postoperative day 3 will be evaluated to examine the effectiveness of the hydrogen gas inhalation treatment. The QoR40 will be measured using a questionnaire on postoperative QOL which will be distributed preoperatively. Each patient will independently fill out the questionnaire himself/herself. The scores will be totaled (perfect score: 200 points), and the resulting number will be evaluated as the patient satisfaction level and recovery level. The questionnaire survey will be conducted at 10 AM on postoperative day 3.

The results of previous reports infer that hydrogen gas inhalation has wide effects on many aspects, such as on inflammation and liver function, by inhibiting ROS. The primary endpoint, therefore, is established as the QoR40 on postoperative day 3. A subinvestigator will record the results of the questionnaire in the case report form after the patient is discharged.

#### Secondary endpoints

Data on the following items will be collected chronologically during the study period and comprehensive and exploratory evaluations will be performed.

1. QoR40s besides that on postoperative day 3
2. Grade of postoperative complications (Clavien-Dindo score): The attending physician will record it in the medical record at discharge.
3. Level of pain (NRS): The attending physician or attending nurse will record it in the medical record after interviewing the patients.
4. Amount of dietary intake: The amount of dietary intake is assessed at breakfast, lunch, and dinner, and 100% is a completely eaten meal. The attending nurse will visually determine the amount of uneaten food to evaluate the amount of dietary intake and will record it in the medical record.
5. Number of pedometer-assessed steps: The number of steps per day will be measured using a pedometer, and the attending nurse will confirm it and record it in the medical record.
6. Inflammatory response (WBC, CRP, neutrophil count, and lymphocyte count): They will be automatically recorded in the medical record.
7. Liver function (T-BIL, AST, ALT, ALP, albumin, platelet count, PT, and ICG): All measurements will be automatically recorded in the medical record.
8. Reactive oxygen level (urinary 8-OHdG* level): *This level reflects the level of oxidative damage to DNA caused by excessive reactive oxygen species. A subinvestigator will measure it and will record it in the case report form.
9. Adverse events: CTCAE version 5.0.

A subinvestigator will enter the aforementioned medical record data in the case report form after discharge.

### Safety evaluation

No clinical trial has previously reported any side effects of hydrogen gas inhalation. In this trial, however, the administration of study gas will be immediately discontinued if any serious adverse event occurs.

All adverse events will be recorded and serious adverse events will be totaled separately. If adverse events occur after study gas administration, they will be totaled separately for group A and group B. In addition, the number of patients who experienced the adverse event, number of adverse event episodes, and incidence of adverse events will be determined, and a comparison will be made between groups A and B. All adverse events will be examined regardless of the causal relationship.

Descriptive statistics will be used for clinical test results for each of groups A and B, and clinically significant changes will be totaled. For other parameters, descriptive statistical values will be calculated by group according to the variable type (continuous or categorical variable), or frequencies will be totaled.

A Data and Safety Monitoring Committee (DSMC) will be established independently from the investigator and sponsor. The DSMC comprises an academic pharmacologist, hepatologist, and hepatobiliary surgeon, and the members will review the unblinded study data or whenever an adverse event is reported. The DSMC will advise on continuing or stopping the study based on safety and efficacy considerations.

### Statistical analysis

For the primary analysis, an unpaired *t*-test with common variance (PPS analysis) will be performed to compare the mean of the QoR40 on postoperative day 3 between groups. The subject population to be analyzed will consist of patients who will have consented to trial participation; who will have been administered, according to schedule, at least the minimum dose of their respective gas for at least 2 days; and whose QoR40 on postoperative day 3 will have been measured. The daily minimum dose will be converted to the duration of administration (3-h duration per day) and will be administered in at least 6 h to less than 7 h. In the sensitivity analysis, a *t*-test will be used on the per-protocol population and similarly on a population which includes patients who received insufficient gas. Content with a focus on the non-unique content will be noted in a statistical analysis plan. No compensation for missing data will be made. The statistical significance level will be set at 5% (two-sided) with a confidence interval of 95% (two-sided). No interim analysis will be performed.

For other items, the effects of hydrogen inhalation will be examined by parameters, such as the extent of hepatectomy (segmentectomy or more extensive resection vs subsegmentectomy or less extensive resection) and hepatic ischemia time (30 min or more vs less than 30 min). In addition, comprehensive and exploratory examinations will be performed, including a comparison of patients with high QoR40 scores and patient characteristics.

### Analysis of efficacy

The measure of the efficacy of the primary endpoint is the improvement of the QoR40 of the per-protocol population on postoperative day 3. The primary subject population for analysis will be the per-protocol population, but analysis of the intention-to-treat population will also be performed to evaluate the potential for practical application.

### Data monitor

All records related to the clinical trial, including source documents, will be available for trial-related monitoring and inspections by the certified review board and regulatory authorities.

Data monitoring will be performed at the centralized data center at Medical Research Support in Japan.

### Dissemination

The results of this study will be prepared for presentation in a peer-reviewed publication.

### Trial status

The trial is proceeding according to the protocol (version 2.0, 19 November 2020). There will be a total of 68 candidates for study subjects with hepatobiliary disease, for which hepatectomy will be planned at the Kansai Medical University Hospital from protocol approval until 31 December 2022 (Table 2).

**Table 2:** Trial data

**Primary registry and trial identifying number**	Japan Registry of Clinical Trial (jRCT): no. 03220332
**Date of registration in primary registry**	Approved on 21 January 2021
**Secondary identifying numbers**	Niigata University Central Review Board of Clinical Research: SP20004
**Source of monetary or material support**	Helix Japan K.K.
**Primary sponsor**	Helix Japan K.K.
**Secondary sponsor(s)**	None
**Contact for public queries**	Masaki Kaibori MD., PhD. Professor Department of Surgery, Kansai Medical University Email: kaibori@hirakata.kmu.ac.jp Postal address: 2-5-1 Shinmachi, Hirakata city, Osaka, Japan. 573-1010 TEL: +81-72-804-0101
**Contact for scientific queries**	Same as above
**Public title**	Effect of inhalation of hydrogen gas on postoperative recovery
**Scientific title**	Effect of inhalation of hydrogen gas on post-hepatectomy recovery Double-blind randomized controlled trial
**Countries of recruitment**	Japan
**Health condition or problem studied**	Hepatobiliary diseases
**Intervention(s)**	Inhalation of hydrogen gas
**Key inclusion and exclusion criteria**	Inclusion criteria: 1. 20 years old and older (regardless of gender) 2. Patient has undergone hepatic resection for hepatobiliary cancer (regardless of the operative procedure, range of hepatectomy, and primary disease) 3. Patient who accepts entry to this study with free will after sufficient explanation Exclusion criteria: 1. Severe liver dysfunction or severe renal dysfunction 2. Uncontrollable diabetes mellitus 3. Pregnancy or possibility of being pregnant 4. Lactating patient 5. Patient who received another test agent or investigational agent within three months prior to agreement for this study 6. Patient who required combined other organ resection (stomach, colon, lung, and so on) 7. Patient who required biliary tract reconstruction 8. Patient who required emergency surgery (HCC rupture) 9. Patient who is considered as inadequate by the principal investigator of this study
**Study type**	Interventional, randomized controlled trial/double blind/placebo or control/parallel assignment/treatment purpose
**Date of first enrollment**	9 March 2021
**Sample size**	68
**Recruitment status**	Recruiting
**Primary outcome**	QoR40 of postoperative day 3
**Key secondary outcomes**	1. QoR40 except postoperative day 3 2. Postoperative complications (Clavien-Dindo score) 3. Wound pain (Numerical Rating Scale) 4. Dietary intake 5. The number of steps taken 6. Inflammatory response (WBC, CRP, neutrophils, lymphocyte) 7. Liver functions (total bilirubin, AST, ALT, ALP, albumin, thrombocyte, PT, ICG) 8. Reactive oxygen value (urinary 8-OHdG) 9. Adverse event 10. Time of inhalation of hydrogen gas
**Ethics review**	Approved Approved number: SP20004 Date of approval: 19 January 2021 Ethics committee: Niigata University Central Review Board of Clinical Research Address: 1-754, Asahimachi-dori, Chuo-ku, Niigata city, Japan, Niigata
**Completion date**	31 December 2023
**Summary results**	No results for now
**IPD sharing statement**	Plan to share IPD: no

*QoR40* the 40-item quality of recovery questionnaire, *CRP* C-reactive protein, *AST* Aspartate transaminase, *ALT* Alanine transaminase, *ALP* Alkaline phosphatase, *PT* Prothrombin time, *ICG* Indocyanine green, *8-OHdG* 8-hydroxy-2’-deoxyguanosine, *IPD* individual clinical trial participant-level data

### Study timeline

Total study period: Japan Registry of Clinical Trial (jRCT) publication day—2023 December 31

Enrollment period: jRCT publication day—2022 December 31

## Data Availability

For protection of privacy of participants, the datasets analyzed during the current study have not been made publicly available, but they can be obtained from the corresponding authors if there are reasonable requirements.
